# Qualitative insights into implementation, processes, and outcomes of a randomized trial on peer support and HIV care engagement in Rakai, Uganda

**DOI:** 10.1186/s12879-016-2156-0

**Published:** 2017-01-10

**Authors:** April Monroe, Gertrude Nakigozi, William Ddaaki, Jeremiah Mulamba Bazaale, Ronald H. Gray, Maria J. Wawer, Steven J. Reynolds, Caitlin E. Kennedy, Larry W. Chang

**Affiliations:** 1 Department of International Health, Johns Hopkins Bloomberg School of Public Health, Baltimore, MD USA; 2 Rakai Health Sciences Program, Rakai, Uganda; 3 Department of Epidemiology, Johns Hopkins Bloomberg School of Public Health, Baltimore, MD USA; 4 Division of Intramural Research, National Institute for Allergy and Infectious Diseases, National Institutes of Health, Laboratory of Immunoregulation, Bethesda, Maryland USA; 5 Department of Medicine, Division of Infectious Diseases, Johns Hopkins School of Medicine, Baltimore, MD USA

**Keywords:** HIV, Pre-ART, Peer support, Randomized controlled trial, Qualitative research, Linkage, Uganda, Mixed methods research, Implementation science

## Abstract

**Background:**

People living with human immunodeficiency virus (HIV) who have not yet initiated antiretroviral therapy (ART) can benefit from being engaged in care and utilizing preventive interventions. Community-based peer support may be an effective approach to promote these important HIV services.

**Methods:**

After conducting a randomized trial of the impact of peer support on pre-ART outcomes, we conducted a qualitative evaluation to better understand trial implementation, processes, and results. Overall, 75 participants, including trial participants (clients), peer supporters, and clinic staff, participated in 41 in-depth interviews and 6 focus group discussions. A situated Information Motivation, and Behavioral skills model of behavior change was used to develop semi-structured interview and focus group guides. Transcripts were coded and thematically synthesized.

**Results:**

We found that participant narratives were generally consistent with the theoretical model, indicating that peer support improved information, motivation, and behavioral skills, leading to increased engagement in pre-ART care. Clients described how peer supporters reinforced health messages and helped them better understand complicated health information. Peer supporters also helped clients navigate the health system, develop support networks, and identify strategies for remembering medication and clinic appointments. Some peer supporters adopted roles beyond visiting patients, serving as a bridge between the client and his or her family, community, and health system. Qualitative results demonstrated plausible processes by which peer support improved client engagement in care, cotrimoxazole use, and safe water vessel use. Challenges identified included insufficient messaging surrounding ART initiation, lack of care continuity after ART initiation, rare breaches in confidentiality, and structural challenges.

**Conclusions:**

The evaluation found largely positive perceptions of the peer intervention across stakeholders and provided valuable information to inform uptake and scalability of the intervention. Study findings also suggest several areas for improvement for future implementation of pre-ART peer support programs.

**Trial registration:**

NCT01366690. Registered June 2, 2011.

## Background

Engagement in care following an HIV diagnosis is imperative to ensure timely initiation of antiretroviral therapy (ART) [1, 2]. Missed clinic visits represent a significant obstacle to the timely initiation of ART [3], and individuals who wait to access care have higher rates of mortality and lower rates of treatment success once they initiate ART [4]. Further, timely initiation of ART may lessen HIV transmission by reducing the viral load of people living with HIV [5–7].

In addition to adherence to clinic appointments and timely initiation of ART, the World Health Organization (WHO) recommends several preventive care interventions for people living with HIV who have not yet started ART (i.e., pre-ART). These interventions include daily use of cotrimoxazole prophylaxis, use of insecticide-treated mosquito nets (ITN) in malaria endemic areas, use of clean water systems, safer sex education, provision of condoms, and psychosocial support [2]. Such interventions can improve quality of life, prevent the continued spread of HIV, delay the progression of HIV, and reduce morbidity and mortality [2].

Despite the demonstrated importance of early engagement in HIV care, many patients in resource-limited settings are unable to initiate and maintain care [8, 9]. Simple and affordable strategies to improve implementation of these proven interventions are therefore urgently needed to improve outcomes among people living with HIV who are pre-ART. Peer supporters – people living with HIV trained to provide basic pre-ART counseling and psychosocial support – may represent one sustainable strategy to address these needs as part of a holistic combination implementation approach to improving HIV care and prevention outcomes [10, 11].

To evaluate the impact of peer support, we conducted a mixed methods study using a two-phase explanatory design [12]. In the first phase, from 2011 to 2013, we conducted a randomized controlled trial (RCT) on the impact of peer support on engagement in HIV care and preventive care utilization [13]. A total of 442 pre-ART, HIV-infected adult participants were randomized to peer support or standard of care and followed for one year. The intervention consisted of monthly structured home visits by peers to intervention arm participants (referred to as “clients”) to provide psychosocial support and promote engagement in HIV care (e.g. attending clinic appointments) and a basic care package of preventive care items including cotrimoxazole prophylaxis, safe water vessel use, insecticide treated bednet use, and condoms.

The RCT had the following key results: participants in the peer support intervention arm were significantly more likely to report being in care, on cotrimoxazole prophylaxis, and adhering to safe water vessel use at 1 year of follow-up. The effect was observed only among care-naive participants, i.e. participants who had never reported being in care prior to being enrolled in this study. The intervention did not clearly impact ART initiation, bednet use, or sexual behaviors.

In the second phase of this mixed methods study, we conducted a post-trial qualitative evaluation to better understand this complex intervention and the accompanying implementation, processes, and outcomes. The results of this qualitative evaluation are reported here.

## Methods

### Study setting

The Rakai Health Sciences Program (RHSP) is a research institution and implementing partner for the U.S. President’s Emergency Plan for AIDS Relief (PEPFAR) in Rakai District, Uganda in East Africa. The post-trial qualitative evaluation was conducted from September to November 2013.

### Conceptual framework

**Fig. 1:**
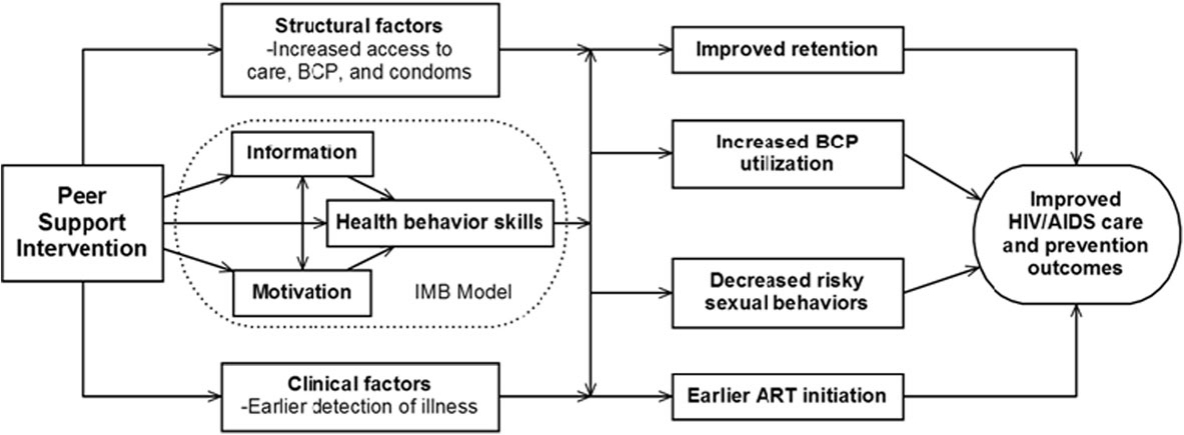
Peer support intervention sIMB-based conceptual framework

The peer support intervention was based on a situated Information, Motivation, and Behavioral Skills (sIMB) conceptual framework of behavior change (Fig. 1) [14]. This model was selected based on the premise that access to information related to HIV treatment and care services, motivation to engage continuously in care, and behavioral skills to navigate the health care system and adhere to preventive treatment measures are key to the understanding and promotion of engagement in care among pre-ART, HIV-infected adults [14, 15]. This model was also felt to be consistent with previous peer-based intervention research in Rakai [16], and had been used in similar contexts evaluating engagement in HIV care [17, 18].

### Qualitative methods

Consistent with a two-phase, explanatory design, qualitative methods were informed by quantitative trial findings. The qualitative evaluation involved in-depth interviews (IDIs) and focus group discussions (FGDs) with 75 total participants. Participants were purposively sampled to ensure representation of perspectives across a range of stakeholders, including people living with HIV from both intervention and control groups, peer supporters, and clinic and RHSP staff.

Semi-structured interview guides were used to explore a number of topics related to the trial including intervention implementation components such as fidelity, context, and reach [19]. IDIs began with open-ended, exploratory questions, then moved to more specific questions related to key sIMB domains and theorized processes of peer support effect on study outcomes.

Forty-one IDIs were conducted with 39 participants (Table 1), including people living with HIV (*n* = 23), peer supporters (*n* = 9) and staff (*n* = 7). Two of the 39 participants, one staff and one peer supporter, completed a follow-up interview in addition to their original interview.

**Table 1:** In-depth interview participants

Category	Males	Females	Total
People living with HIV	9	14	23
Intervention	5	10	15
Control	4	4	8
Peer Supporters	3	6	9
Program staff	3	4	7
Total	15	24	39

IDIs provided the opportunity for respondents to speak freely about their experiences with the peer support intervention. IDIs were conducted in three phases to ensure an iterative process in which initial findings, discussed by multiple team members, could inform future rounds of data collection. This strategy enabled adaptation of field guide questions and expansion of initial findings through more detailed exploration of emerging topics.

After three rounds of IDIs, FGDs were conducted to explore group discussion around specific topics form the IDIs. Six mixed-gender FGDs were conducted, ranging from 5 to 8 participants each, with a total of 36 participants. Five of these FGDs were held with clients in the intervention group (*n* = 30) and 1 FGD was held with peer supporters (*n* = 6). Clients in the FGDs were different individuals from those selected for participation in IDIs. Peer supporters were allowed to participate in both IDIs and the FGD due to the small number of total peer supporters in the study and differences between the nature of information gathered in the personal context of an IDI compared to the group dynamic of an FGD.

FGDs were led by members of the RHSP qualitative research team and were used to clarify and expand on themes identified in the IDIs. Discussion, agreement, and dissent that arose in the focus group setting facilitated a process of “sharing and comparing” that led to a more nuanced and comprehensive understanding of the peer support intervention [20].

All IDIs conducted in English were recorded and transcribed verbatim, while IDIs and FGDs conducted in Luganda, the local dialect, were recorded, directly translated into English, and transcribed.

A preliminary codebook was developed both deductively, through review of field guides, research questions, and inductively, through identification of new themes from IDI and FGD transcripts. Transcripts were uploaded into ATLAS.ti Scientific Software [21] for ease of storage, indexing, and retrieval. The preliminary codebook was used to index a random sample of 10 transcripts, and the team made adjustments until a complete codebook was created. The complete codebook was used to index all transcripts, including those used in the preliminary coding exercise. We followed a thematic approach to data analysis [22]. Data was synthesized by theme to identify common patterns from responses of participants, and the coded text was analyzed to identify further themes and patterns for interpretation.

## Results

Deductive analysis found that results organized into several themes. Foremost, the sIMB model-based conceptual framework proved relatively robust and helped provide insights into study and intervention implementation and processes, including highlighting a number of implementation challenges. A number of explanatory themes also emerged regarding trial outcomes. Results are organized sequentially as follows: sIMB model domains (information, motivation, behavioral skills, and situated factors), implementation challenges, and insights into quantitative trial findings.

### Information

Information relates to knowledge of the purpose of attending HIV care, the system of care, and the characteristics of care interventions [14]. People living with HIV in both the intervention and control groups reported receiving information on HIV care, treatment, and prevention from a variety of sources, including mass media and health workers.

Clients in the control group noted the importance of having information accessible in the community and brought directly to people living with HIV. One control group client discussed the challenges faced by people in his community who lack knowledge about HIV services. When discussing the potential value of a peer support program he stated:

“The advantage with it is that there are people out there who are not familiar with what is going on. They keep to themselves to a point when they become weakened so much. If these people (peer supporters) visit him and share with him what is going on, especially telling him about the current treatment, they can tell him to come for treatment, learning how he should behave and use what he has been given. For sure he will benefit a lot from it.” **(IDI-Client-Control)**

In the intervention arm, clients were able to benefit from peer supporters reinforcing health information and helping to better explain complicated health messages to clients. For example, an intervention group client described how her peer supporter reinforced and further clarified information she was given at the clinic:

“The peer [supporter] helps to interpret some things for me that I may not have understood when the health worker was telling me. The health workers can give drugs or blood results that I may not understand but when the peer [supporter] visits me she explains to me each and everything well and I understand whatever she tells me…” **(IDI, Client-Intervention)**

### Motivation

In the sIMB model, motivation involves both personal and social motivation [14]. On a personal level, motivation to use HIV care and to focus on longevity and enhanced quality of life have been linked to engagement in care. Social motivation can include involvement of family, friends or community members in supporting HIV care or reducing HIV stigma [14]. Qualitative findings revealed that many clients in both the intervention and control groups felt motivation to adhere to care. When asked what enables her to keep her clinic appointments, one female control group client explained: “I want to help my children grow up because they are still young. So I have to take that responsibility to take my drugs, take them on time and respect my clinic dates.” **(IDI- Client-Control)**

In many cases, the peer support intervention enhanced both personal and social motivation among clients. The process through which the intervention impacted motivation appeared to be through development of support networks and promotion of optimism and living positively. A male peer supporter described how he talks to his clients about the future:

“I tell them that the issue of having HIV is not the end of them. It is now getting to the rainy season. I tell them to plant enough food and encourage them to plant coffee trees for money. Some may develop a feeling that it is useless to plant coffee trees because they take long before they mature and they are going to die. We continue to teach them that it is not the end of life.” **(IDI, Peer Supporter)**

Intervention arm participants described the encouragement that peers gave clients, the persistence peers showed in helping clients, and the ways in which peers went beyond their assigned responsibilities to ensure client success. This included introducing clients to other people living with HIV and talking to spouses and other family members when necessary to ensure that the client received the support he or she needed at home. A male peer supporter explained:

“There was one client of mine who had not disclosed [her HIV status] to her husband because she feared that her husband would chase her away. So she told me and I also told the [RHSP] counselor and we went together to her place to talk with the man. The man talked to us nicely and he had to accept to stay with his wife and they are living happily now.” **(IDI, Peer Supporter)**

Several participants echoed the concept of peer supporters providing clients with hope and a positive outlook on life. One client from the intervention group discussed this topic as follows:

“I feared a lot and said I will not go back to the clinic to get the drugs the second time. She told me no, you have to continue going to the clinic for drugs. She told me that you are now the head of the house and you have to take care of the children…She told me not to lose hope because I was not going to die…That is how she encouraged me.” **(FGD, Client-Intervention)**

### Behavioral skills

Behavioral skills relate to one’s ability to navigate the health system, make and remember appointments, garner social support, correctly take medications, and use condoms [14]. Both control and intervention clients described behavioral skills they had developed during the study.

The process through which peer supporters improved behavioral skills among clients included teaching them how to navigate the health system, encouraging them to pay attention to their number of pills and clinic appointment dates, and developing strategies aimed at improving engagement in care, such as attending with a friend who has an appointment on the same day.

A female peer supporter discussed a strategy to help some of her young male clients to remember to take their cotrimoxazole every day by making it a part of their morning routine:

“Young men are too forgetful…I encourage them to take the drugs in the morning. ‘After waking up and washing your face take your drugs with enough water or even a full cup of water. But if you don’t swallow the drug and go for garden work or any work, you get deals and don’t come back home in time to take the drugs.’” **(IDI, Peer Supporter)**

### Situated factors

Situated factors in the sIMB model refer to structural (e.g. supply chain resiliency), clinical (e.g. patient’s health status), and environmental (e.g. access to care) factors which may have a role in explaining intervention processes and effects, i.e. context [23]. For example, peer supporters and clients often reported that mobility was a significant barrier, both to receiving care and treatment and to successfully implementing home visits, particularly in fishing communities with highly mobile populations.

Another structural challenge frequently reported was supply shortages. A number of people living with HIV reported only receiving cotrimoxazole, rather than the full package of preventive care items, due to shortages in the supply of ITNs and water vessels. One program staff member voiced the structural challenge as follows:

“So, such things have happened in the supply chain… at a close of a year, before next funding is awarded, there can be some interruptions in the supply chain. …Unfortunately it’s not within our control to prevent.” **(IDI, Staff- Program)**

Clients, peer supporters, and staff frequently discussed how peer supporters went beyond their defined role to help overcome health system challenges, resulting in improved access to and quality of care. Many peer supporters took on clinic-based roles and responsibilities, which was not included as part of the intervention. Participants described how peer supporters helped organize clinic sessions and acted as a bridge between clinic staff, patients, families, and community members, strengthening structural links in the health care system and providing clients with opportunities to more effectively navigate and access the health care system. One female clinic staff explained how peer supporters helped to overcome time constraints she experienced in the clinic:

“Peer supporters, they do a lot of work, they help me a lot…if you are a nurse, you work very hard, you find you are very busy with the patients, and if you go and try to look out for a certain patient, you will find the time is not allowing. But a peer can come… and you can tell them, ‘there is this person that I need, can you pick him for me?’” (**IDI, Staff-Clinic)**

A male peer supporter discussed helping clients to overcome transportation challenges as follows: “If this person has no means of transport to reach the health facility, you can ride him/her on a bicycle and if you have a boda boda (motor cycle) you can ride it and take this person to the health facility for treatment.” **(In-depth interview, Peer Supporter)**

Peer supporters also described working together to provide care to mobile clients through sharing information and ensuring their clients had access to support. A male peer supporter explained:

“We peer supporters coordinate very well and at least we have each other’s phone contact. So if I reach a patient’s place and I find when he/she has shifted to another community, I can call the peer supporter of that community to help that patient and I can give him/her more details about this patient.” **(IDI, Peer Supporter)**

### Challenges and areas for improvement

The qualitative evaluation identified a number of challenges with the peer support intervention. Peer supporters and clients frequently identified ART knowledge as an area in need of improvement. Clients raised questions and concerns about ART which peer supporters were not able to adequately address as their training and responsibilities were limited to pre-ART issues.

While reported by only a small minority of participants, another important challenge was maintaining client confidentially, specifically preventing inadvertent disclosure of clients’ HIV status. This was not due to deliberate peer supporter malfeasance but rather the peer supporter would sometimes wear a “uniform” identifying them as a peer supporter and become known for visiting people living with HIV. Some strategies suggested by study participants to minimize unplanned disclosure included notifying clients by phone prior to making a home visit, coming like any other visitor (e.g., wearing normal clothes rather than a peer supporter t-shirt), meeting in private, and abstaining from leaving messages with neighbors or family members unless given explicit consent by the client. Many peer supporters reported already using these strategies to protect confidentiality:

“The things I was going to use were well arranged. The papers were in place, I arrived home okay they tell us that we go on foot but I have a motorcycle. I put my bag on the motorcycle. You go to them like any other visitor. You know there can be some other people at home who should not know what takes place. I wait to be welcomed and talk to them about the purpose of my visit. When they accept to meet me, then I go back to my motorcycle pick my stuff and we discuss.” **(IDI, Peer Supporter)**

The importance of contacting clients by phone prior to meeting was recognized across clients, peer supporters, and staff; however, many peer supporters reported the challenges associated with visiting clients who did not have a mobile phone. This issue compounded concerns over transportation, with peer supporters emphasizing the challenge of arriving for a home visit and not being able to locate the client.

Finally, peer supporters reported that some clients did not wish to be visited. Though all clients had consented to be in the study with the understanding that a peer might visit them at home, some clients remained hesitant when first approached by a peer. Peer supporters were often able to establish productive relationships with clients over time, but there remained a small and difficult to engage segment of the population.

### Trial insights

In addition to providing a better understanding of intervention implementation processes and mechanisms of effect, qualitative findings also provided insights into quantitative trial results as described below.

#### Increased engagement in care

The trial’s finding that peer support increased engagement in HIV care was supported by results from the qualitative evaluation. A staff member explained the ability of peer supporters to improve engagement in care as follows:

“Normally I look at the HIV care journey, so from diagnosis to a life-long treatment or care, as a journey where you then need an encourager. Sometimes our patients battle with discouragement, depression, and I think anyone who can hold your hand along that journey can be helpful.” **(IDI, Staff-Program)**

Intervention arm participants consistently reported receiving information regarding the benefits of attending clinic; feeling increased motivation associated with improved self-confidence, family and community support, and reduced stigma; and having the behavioral skills necessary to remember and attend appointments.

#### Increased use of cotrimoxazole prophylaxis; no difference in ART initiation

Qualitative findings revealed potential explanations for this finding that could not be derived from quantitative analysis alone. Clients noted the quality and quantity of information provided about cotrimoxazole differed from that provided about ART. Peer supporters provided a large amount of information and encouragement around taking cotrimoxazole compared to ART. Peer supporters regularly provided information on the benefits of cotrimoxazole, enquired about use, and reminded clients to take it.

Some participants indicated that the focus on staying on cotrimoxazole for as long as possible may have led to negative views and fear surrounding ART initiation among some clients. One client from the intervention group explained:

“When you talk with the peer supporter they tell you to be active in taking cotrimoxazole because antiretrovirals are so strong. When you are told that you are going to begin antiretrovirals your heart trembles. You ask yourself how you are going to begin it. So you can fear that drug and it can bring such problems.” **(FGD, Client-Intervention)**

In contrast, participants mentioned that when clients initiated ART, their peer supporters were no longer tasked to visit them due to the emphasis of the intervention on pre-ART care. Some participants indicated this was a disincentive to start ART due to the high value clients placed on their relationship with their peer supporter. This unexpected finding may help explain the lack of difference in ART initiation between the two groups.

#### Increased water vessel use; no difference in bednet use

A reduction in stigma may help explain results that showed that participants visited by a peer supporter were more likely to use the clean water vessel [24]. The qualitative evaluation revealed that the water vessels were viewed as stigmatizing by a number of people living with HIV because they were different from other water vessels in the area and were specifically associated with HIV. A male client explained:

“We are told that all the information about us is kept so confidential. The water vessel was abandoned because it is identified with persons with HIV. Okay, I disclosed, accepted and I am satisfied with my condition of having HIV but that water vessel has been identified with HIV. So every family you find that water vessel, it is known, there is someone with HIV.” **(IDI, Client-Intervention)**

The stigma associated with the water vessel may explain why overall water vessel use was low among clients. Peer supporters, however, were able in some cases to overcome this barrier though education, encouragement, and teaching strategies for dealing with stigma.

In contrast, bednets did not appear to be associated with HIV status in the Rakai community. As a result, promotion of their proper use was not strongly viewed as an HIV care intervention and not affected by clients’ experiences with stigma. This perception may explain why bed net use was high across study arms.

#### No difference in sexual behaviors

Qualitative results showed contrasting views on what impact peer support might have had on clients’ sexual behaviors. Some participants noted that clients in the peer support intervention generally felt better physically and mentally because they were taking their cotrimoxazole, and therefore were more likely to be sexually active and considered sexually attractive. However, some participants insisted the opposite, that clients in the intervention arm were influenced by peer supporter counseling to reduce their number of sexual partners or increase condom use. These potentially contrasting effects may have contributed to the lack of a difference in sexual behaviors [24].

Additionally, women may be unwilling or unable to insist upon condom use due to gender norms, threats of violence, or broader perceptions that those who use condoms are promiscuous. A program staff member described the cultural and gender-based barriers to condom use in the following way:

“Traditionally, the female is the weaker sex, so females in the African tradition may not bargain for safe sex as effectively, without disrupting their marital relationship. So that becomes a challenge, having a woman convince a man to wear a condom. So, and many times, unless the man takes it up as his responsibility, it’s likely to fail because the woman does not have a big say in actually persuading him to use a condom.” **(IDI, Staff).**

Others noted that some people living with HIV are involved in sex work, and therefore their livelihood is tied to specific sexual behaviors, which were difficult to change.

#### Contamination of control arm

One possible explanation for null results for some study outcomes, and/or lack of a larger effect for positive results, may be contamination of the control arm. Peers were tasked only to support their assigned clients, but they sometimes expanded upon their official roles. For example, peer supporters at times addressed a need for information and support at the community level, providing services to community members beyond their own clients. A male peer supporter explained:

“Since I became a peer supporter I relate with very many people in the community and even with the health workers. Being a peer supporter has helped me to get very many friends because even those people who are not my patients, the moment they see me holding my files, they try to consult me and ask me a lot of things about their health and I do advise them to go for HIV testing to know their HIV status if they have not yet tested for HIV.” **(IDI, Peer Supporter)**

While this was not a common theme, peers providing direct support to control arm clients or impacting them indirectly through reducing community-wide stigma may have made it more difficult to detect small effects of the peer support intervention.

## Discussion

The sIMB framework provided a valuable lens for understanding client behaviors related to engagement and adherence to care and the processes through which the peers impacted these behaviors. Peer supporters helped clients make sense of confusing health information, provided psychosocial support, helped navigate the health care system, and improved relationships with family, friends, and their community. These positive findings also helped explain mechanistically how the intervention impacted study outcomes such as engagement in care and use of cotrimoxazole.

The qualitative evaluation also illuminated the importance of the situated element of the sIMB model, which underscores the importance of looking at the context in which HIV care is delivered and the structural factors that may promote or impede care utilization. For example, supply of mosquito nets and safe water vessels needed to be improved at the clinics, and lack of availability may have negatively impacted the ability of the trial to fully assess the peer support effect on these outcomes.

A critical challenge identified in this study was inadvertent HIV serostatus disclosure. Along with the suggestions offered by study participants, future interventions may mitigate this issue by including both HIV-infected and HIV-uninfected persons. This approach may also reduce the stigma associated with peer home visits and help engage difficult to reach populations. Finally, an approach addressing the entire HIV care continuum that is not limited to just pre-ART, but also includes people living with HIV on ART, could help resolve some issues caused by a solely pre-ART program.

Finally, this study generated several potential explanations for trial findings. Positive trial outcomes were well reflected in qualitative results, and null trial outcomes were better understood through context provided by qualitative study findings. For example, the very low uptake of water vessel use by people living with HIV was plausibly explained by association of water vessel use with being HIV-positive.

The qualitative evaluation made use of an iterative process to obtain rich information on the intervention and utilized multiple research methods to triangulate information. Despite these strengths, the study had a number of limitations. While participants were encouraged to speak freely about the peer support program, it is possible that peer supporters and clients in the intervention group withheld negative perceptions of the program in order to provide responses they felt would be socially acceptable. Similarly, program staff was included as participants in the qualitative evaluation, and may have had an interest in presenting the intervention in a positive light. However, the consistency of findings across participants, combined with open discussions of challenges and frustrations associated with the program seem to indicate that these factors did not significantly influence findings. Also, while the sIMB model is broad in nature and has been found to be a useful tool for understanding engagement in HIV care, structuring the study design around the sIMB model may have limited identification of additional factors related to the outcomes of interest. While an important consideration, this issue was mitigated through use of open-ended questions and by providing opportunities for unstructured comments by participants.

This qualitative evaluation was also not able to probe specifically into the trial result that found that the impact of the peer support intervention was only in care-naïve participants, as this finding was not discovered until after the qualitative evaluation was complete. However, the qualitative evaluation did suggest that there was substantial variation in clients’ levels of knowledge, motivation and behavioral skills within control and intervention groups. It is possible that clients already engaged in care and using cotrimoxazole, i.e. care-experienced at baseline, may have already had many of the information, motivation, and behavioral skills needed to engage in care.

Despite these limitations, this qualitative post-trial evaluation generated knowledge which complemented trial findings and provided important insights into implementation, processes, and outcomes of a peer support intervention promoting HIV care engagement.

## Conclusions

This post-trial qualitative evaluation provided valuable complimentary findings to the quantitative results from a randomized trial. The evaluation found largely positive perceptions of the peer intervention across stakeholders, suggested that intervention implementation largely followed the original conceptual framework, and provided plausible explanations for trial outcomes. Findings also suggested several areas for improvement for future implementations of pre-ART peer support programs.

Our study revealed important dynamics related to the interactions between peer supporters and clients, staff, and the community, thus contributing to a better understanding of particular intervention mechanisms, intended or unintended, that were important to the study outcomes. By combining qualitative and quantitative results, a clearer picture was formed of the potential value peer supporters can play in improving engagement in care for this important population.

## Abbreviations

ART: Antiretroviral therapy
FGD: Focus group discussion
HIV: Human immunodeficiency virus
IDI: In-depth interview
ITN: Insecticide-treated mosquito net
PEPFAR: U.S. President’s Emergency Plan for AIDS Relief
RCT: Randomized controlled trial
RHSP: Rakai Health Sciences Program
sIMB: Situated information, motivation, and behavioral skills
WHO: World Health Organization
